# The activation of pyrin domain-containing-3 inflammasome depends on lipopolysaccharide from *Porphyromonas gingivalis* and extracellular adenosine triphosphate in cultured oral epithelial cells

**DOI:** 10.1186/s12903-015-0115-6

**Published:** 2015-10-29

**Authors:** Wei Guo, Peng Wang, Zhonghao Liu, Pishan Yang, Ping Ye

**Affiliations:** Department of Periodontology, Shandong Provincial Key Laboratory of Oral Biomedicine, Shandong University, Jinan, 250012 People’s Republic of China; Department of Endodontics, Yantai Stomatological Hospital, Yantai, Shandong Province China; Yantai Stomatological Hospital, Yantai, Shandong Province China; Institute of Dental Research, Westmead Millennium Institute and Westmead Centre for Oral Health, Westmead Hospital, Westmead, Australia

**Keywords:** Extracellular adenosine triphosphate (ATP), NLRP3 inflammasome, *Porphyromonas gingivalis* (*P. gingivalis*), *P. gingivalis* LPS

## Abstract

**Background:**

Gingival epithelial cells are the major population of the gingival tissue, acting as the front-line defense against microbial intrusion and regulating the homeostasis of the periodontal tissue in health and disease via NLR family pyrin domain-containing-3 (NLRP3) inflammasome, which recognizes pathogen- and danger-associated molecular patterns (PAMPs and DAMPs). The aim of this study was to determine whether the activation of NLRP3 inflammasome depends on infection with the periodontal pathogen *Porphyromonas gingivalis* (*P. gingivalis*), or stimulation with *P. gingivalis* lipopolysaccharide (LPS), and/or extracellular adenosine triphosphate (ATP).

**Methods:**

An oral epithelial cell line was treated with *P. gingivalis*, *P. gingivalis* LPS and ATP. The gene and protein expression of NLRP3 inflammasome components were quantified by real time RT-PCR and immunoblots. Production of IL-1β and IL-18 was measured by ELISA.

**Results:**

There was no increase in NLRP3 inflammasome gene expression after *P. gingivalis* infection unless pre-stimulated by ATP. Obvious increases of NLRP3 inflammasome gene expression was observed after *P. gingivalis* LPS stimulation, even pre-stimulated by ATP at 2 h.

**Conclusions:**

The findings indicate that the activation of NLRP3 inflammasome does not rely on *P. gingivalis* infection, unless stimulated by *P. gingivalis* LPS and/or extracellular ATP, suggesting diverse signaling pathways are involved in the host immune response.

## Background

Chronic periodontitis, one of the most common and prevalent diseases in humans worldwide [1], is defined as an infection-driven chronic inflammatory disease of the periodontium resulting in the destruction of gingival tissue, absorption of alveolar bone and eventually tooth loss [2]. When infection occurs, the innate immune system will be the first line defense against pathogens and activates the adaptive immune system for sustained protection from such invasions [3].

The intracellular multi-protein complexes, known as “NLR inflammasomes” play a central role in innate immunity. Among inflammasomes, the pyrin domain-containing-3 (NLRP3) inflammasome is the most studied [4]. Gingival epithelial cells express a functional NLRP3 inflammasome that can be activated by pathogen- or danger-associated molecular patterns (PAMPs or DAMPs) [4]. NLRP3 is assembled with the adaptor protein ASC (apoptosis-associated speck-like protein) into a multi-protein complex that governs caspase-1 activation and subsequent maturation the of pro-inflammatory cytokines interleukin (IL)-1β and/or IL-18 in the host response to periodontal infection [4]. Gingival epithelial cells can sense and recognize PAMPs or DAMPs [5] through stimulation of pathogen recognition receptors (PRRs) [1, 3], with subsequent release of the pro-inflammatory cytokines IL-1β and IL-18 [6]. The fundamental work by Bostanci et al. [7] first indicated that NLR inflammasomes are involved in periodontal disease,which responses to *P. gingivalis* infection both clinical and *in vitro* studies.

Interleukin-1β (IL-1β), a proinflammatory cytokine belonging to the IL-1 family, is critical in the host defense against microbial infections [5] and regulates innate immune and inflammatory responses. IL-18 is another proinflammatory cytokine belonging to the IL-1 family [6] and has recently been described as an important element in the inflammasome system that activates caspase-1 and leads to the activation of the inflammation process. Recent evidence has demonstrated that the maturation and secretion of IL-1β and IL-18 are regulated by the NLRP3 inflammasome complex which contains the NLRP3 scaffold, caspase-1 and apoptotic speck protein containing a C-terminal caspase recruitment domain (ASC) [8]. Excess IL-1β and IL-18 contribute to an increasing number of human inflammatory responses [9]. Although IL-1β and IL-18 belong to the same cytokine family, their gene expression and secretion are differentially regulated in human monocytic cells in response to *P. gingivalis* [10]. Therefore, cytokines of the IL-1 family may participate *via* different pathways in the complex pathogenesis of periodontitis [10].

*P. gingivalis*, a gram-negative anaerobic bacterium [11], has been confirmed to be a predominant periodontal pathogen [12] and produces a number of potential virulence factors to perturb the host defense system [13] and induce an inflammatory response in periodontal diseases [14]. However, the effect of *P. gingivalis* on activation of the NLRP3 inflammasome remains controversial. Lipopolysaccharide (LPS), as the major cell wall component of *P. gingivalis* [15], is considered an important virulence factor eliciting the inflammatory response in the periodontal disease [16]. It seems to be unanimously agreed that LPS treatment of mononuclear phagocytes, [17] macrophages [18] and oral epithelial cells [19] significantly induces the expression of NLRP3 and procaspase-1 at both the mRNA and protein levels. Studies have shown that any functional polymorphism in LPS-receptors affects the inflammatory process and the clinical outcomes of periodontal disease [20]. The findings outlined above suggest that whole *P. gingivalis* and *P. gingivalis* LPS may have different effects on the activation of NLRP3 inflammasome system.

Extracellular ATP (adenosine triphosphate), one of the first activators described to induce NLRP3 inflammasome formation, is ascribed to the group of endogenous DAMPs released by dying or injured cells [21, 22]. Its presence is negligible in healthy tissues, but may rise to high micromolar levels following tissue damage at sites of inflammation [23]. Studies have shown ATP induced caspase-1 activation and subsequent IL-1β release [24, 25]. Furthermore, Özlem et al. demonstrated that IL-1β was not secreted unless LPS-treated or infected gingival epithelial cells (GECs) were subsequently stimulated with ATP, and that ATP had no additional effect on NLRP3 or ASC expression in *P. gingivalis* infected GECs [26]. These results point out the need to further evaluate the role of ATP in NLRP3 inflammasome activation.

Therefore, the activation of the NLRP3 inflammasome complex in a cultured oral epithelial cell model by *P. gingivalis* infection, or *P. gingivalis* LPS, or ATP stimulation was tested. This provided further understanding of the mechanism behind periodontal inflammation.

## Methods

### Oral epithelial cell culture

The epithelial cell line (H413) derived from a human oral squamous cell carcinoma [27], displays stratified epithelial cell morphology in culture. H413 cloned cell lines were established using a limit dilution method as described previously [28]. The cloned cells were cultured in Eagle’s Minimum Essential Medium (JMEM, Joklik modification, Sigma-Aldrich, St Louis, MO, USA), penicillin/streptomycin (100 IU/ml, Sigma) and 10 % fetal calf serum (FCS, CSL Limited, Victoria, Australia) at 37 °C in 5 % CO_2_ [29]. Cultures were harvested with triple express (replacement for trypsin, Invitrogen, Life Technologies, Carlsbad, CA, USA) in PBS and sub-cultured every 3 days.

### Bacterial cell culture

*Porphyromonas gingivalis* (ATCC 33277) was cultured anaerobically for 24 h at 37 °C in a trypticase soy broth supplemented with haemin (5 mg/ml, Sigma) and menadione (1 mg/ml, Sigma). On the day of cell treatment, bacteria were centrifuged at 5000 rpm, and 4 °C for 15 min, washed twice and re-suspended in cold PBS, pH 7.3.

### Cell treatment

Confluent H413 cell cultures (5 × 10^6^ cells in T-25 cm^2^ flasks) were washed three times with PBS and infected with *P. gingivalis* at a multiplicity of infection (MOI) of 100 bacterial cells per one epithelial cell [30] for 2 and 4 h [31, 32], or stimulated with 1-μg/mL ultrapure lipopolysaccharide (LPS) from *P. gingivalis* (Invivogen, San Diego, CA, USA) in the cell growth media for 2 and 4 h. Uninfected and non-stimulated cells served as controls.

Experiments were also carried out after pre-incubating the cells with 5 mM adenosine triphosphate (ATP) (Invivogen) for 3 h prior to infection with *P. gingivalis* or stimulation with *P. gingivalis* LPS for 2 and 4 h. Cells pre-incubated with 5 mM ATP for 3 h were as controls for these groups.

### RNA isolation and quantitative real-time RT-PCR

After treatment, cells were harvested in 1 ml of Trizol reagent (Invitrogen) and RNA extracted as per the Trizol protocol. For reverse transcription, the First-Strand cDNAs were synthesized with oligo (dT)_12–18_ (Invitrogen), 10 mM dNTP (Promega, Madison, WI, USA), 5× first stand buffer, RNaseOUT™ Recombinant RNase Inhibitor (Invitrogen) and SuperScript™ III Reverse Transcriptase (Invitrogen) according to the manufacturer’s protocol.

Primers for genes encoding inflammasome components (NLRP3, ASC and caspase-1) and the cytokines IL-1β and IL-18 (Table 1) were designed using Oligo Explorer software (1.1.0) and synthesised by Integrated DNA Technologies (IDT, Coralville, IA, USA). Real-time RT-PCR analyses were performed by SYBR Green based assays using the Stratagene MxPro-Mx3005P System (Agilent Technologies, Santa Clara, CA, USA). PCR reactions were conducted with 2 μl of diluted cDNA samples, 200 nM of each respective forward and reverse primer in a 20 μl final reaction mixture with Platinum SYBR Green qPCR SuperMix-UDG (Invitrogen). cDNA samples isolated from non-manipulated H413 clone-1 cells were quantified by PicoGreen kit (Invitrogen) and then used for constructing standard curves (2–2000 pg) by reference to the expression of the house keeping gene encoding β-actin. The PCR reactions for each gene were carried out in triplicate in 96-well plates, and initiated by activation at 95 °C for 2 min, followed by 40 PCR cycles of denaturation at 95 °C for 15 s, annealing and extension at 60 °C for 30 s. The results were analysed using MxPro 4.10 software.

**Table 1:** Primers used for real-time RT-PCR

Genes	Oligos	Primers5′-3′	Expected amplicon size	UniGene numbers
NLRP3	F-primer	GCTGGACCTGAGTGACAAC	151 bp	Hs.159483
	R-primer	GCTGAGTACCGAGGACAAAG		
ASC	F-primer	AGGCCTGCACTTTATAGACC	174 bp	Hs.499094
	R-primer	GCTGGTGTGAAACTGAAGAG		
caspase-1	F-primer	GAAAAGCCATGGCCGACAAG	205 bp	Hs.2490
	R-primer	GCCCCTTTCGGAATAACGGA		
IL-1β	F-primer	GGCCCTAAACAGATGAAGTG	90 bp	Hs.126256
	R-primer	GTAGTGGTGGTCGGAGATTC		
IL-18	F-primer	GCATCAACTTTGTGGCAAT	161 bp	Hs.83077
	R-primer	CCGATTTCCTTGGTCAAT		
β-actin	F-primer	ACTCTTCCAGCCTTCCTTC	216 bp	Hs.520640
	R-primer	GGAGCAATGATCTTGATCTTC		

### Immunoassay-ELISA to quantify levels of IL-1β and IL-18

A standard sandwich enzyme-linked immuno-sorbent assay (ELISA) was used to measure cytokine production of IL-1β and IL-18. Briefly, supernatants of test (treated with *P. gingivalis*, *P. gingivalis* LPS, ATP+ *P. gingivalis* or ATP+ *P. gingivalis* LPS) and control cell cultures were collected at each hour (1, 2, 3, 4, 5, 6 h), then particles removed by centrifugation and analysed immediately or aliquoted and stored at −20 °C. Human IL-1β and IL-18 specific monoclonal and polyclonal antibodies (1 μg/ml) were pre-coated onto high binding 96-well plates (Corning Incorporated, USA) in carbonate buffer (pH 9.0) at 4 °C overnight. After removing the coating solution and washing the plate three times with 200-μl PBS/well, with the plate was blocked with 3 % bovine serum albumin (BSA, Sigma) at 4 °C overnight. The test samples were added to triplicate wells for 90 min at room temperature, and followed by washing with 0.05 % Tween20/PBS (TPBS) three times; then incubated with secondary antibody (goat-anti mouse/rabbit IgG, (DAKO, Glostrup, Denmark) conjugated with alkaline phosphatase (AP)) diluted 1:1500 in TPBS for 2 h at room temperature then washed with TPBS buffer 3 times. Bound conjugates were detected by pNPP (p-Nitrophenyl-phosphate) and the absorbance measured at 405 nm in a microplate reader (Bio-Rad, Hercules, CA, USA) after 15–30 min incubation at room temperature. Reactions were stopped by adding an equal volume of 1.00 M NaOH. The human IL-1β and IL-18 concentration of the samples were interpreted from a standard curve.

### Immunoblots for NLRP3, ASC and caspase-1 proteins

To measure inflammasome protein expression, 2- and 4-h cultures of the different conditions (treated with *P. gingivalis*, *P. gingivalis* LPS, ATP 3 h + *P. gingivalis* or ATP 3 h + *P. gingivalis* LPS) were extracted in SDS sample buffer and separated by PAGE using gradient 5 to 12 % mini-gels, transferred to nitrocellulose membranes (Bio-Rad) and blocked overnight with 3 % BSA (Sigma) in 0.1 M Tris buffered salts solution pH 7.4 (TBS). Blotted antigens were incubated with rabbit polyclonal anti-human antibodies CIAS1/NALP3, TMS1/ASC, Caspase-1 (1 μg/ml, Abcam, Cambridge, UK), and β-actin (0.1 μg/ml, GenTex, Zeeland, MI, USA) as a loading control in 0.05 % Tween20/TBS for 4 h, washed three times and subsequently incubated with alkaline phosphatase (AP)-conjugated secondary antibody (goat-anti rabbit IgG, DAKO) diluted 1:1500 in Tween20/TBS for 2 h. Bound antibody was visualized with AP substrate (Bio-Rad) after development of reactivity for proteins from control antibody.

### Statistical analysis

All data were analysed by paired *t*-test (mean ± S.D., two-tailed, 95 % CI range) from at least three consecutive experiments for real-time RT-PCR and ELISA. For western blot quantification, the densitometric analysis was performed on the grey level intensity of target bands relative to control β-actin bands derived from scanned films, processed by using Gene Tool image analysis software (GeneToos, version 4.02; Syngene, Cambridge, UK) [33]. *P* < 0.05 was considered statistically significant.

## Results

### Inflammasome (NLRP3, ASC and caspase-1) expression in response to *P. gingivalis*, *P. gingivalis* LPS and ATP plus *P. gingivalis*/*P. gingivalis* LPS

In all experiments, there were no significant differences in gene expression between unstimulated control groups and control groups pretreated with ATP.

**Fig. 1:**
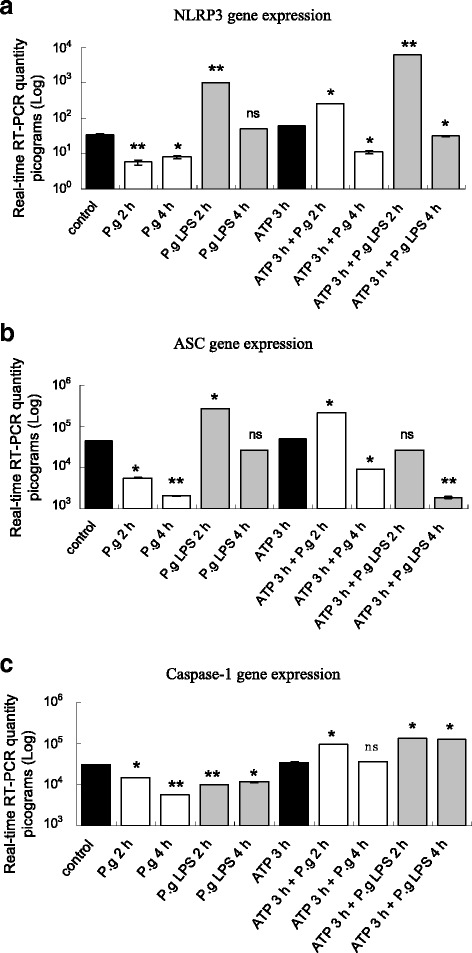
Gene expressions of NLRP3、ASC and caspase-1. Significant changes in inflammasome genes encoding NLRP3 (**a**), associated adaptor protein (ASC) (**b**), and caspase-1 (**c**) with different treatments of *P. gingivalis* infection, *P. gingivalis* LPS stimuli, and ATP plus *P. gingivalis* or *P. gingivalis* LPS in H413 epithelial cells. * *P* < 0.05, ** *P* < 0.01, paired *t*-test

**Fig. 2:**
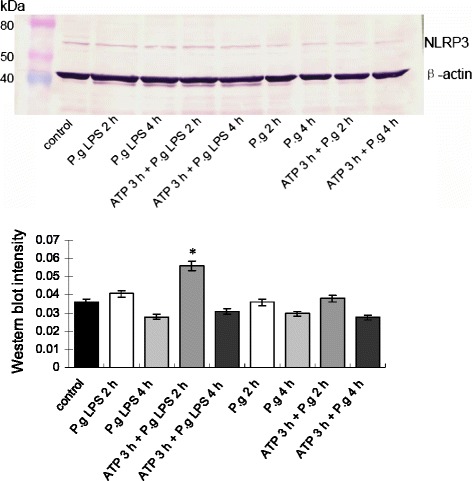
Western blot showing NLRP3 protein expression in H413 epithelial cells treated with different conditions (*P. gingivalis*, *P. gingivalis* LPS, and ATP plus *P. gingivalis* or *P. gingivalis* LPS). The trends of the measured NLRP3 bands corresponded to NLRP3 gene expression. * *P* < 0.05, paired *t*-test

As shown in Fig. 1a, there was down-regulation of NLRP3 gene expression after *P. gingivalis* infection at 2 and 4 h compared with the control group. In contrast, there was increased gene expression of NLRP3 at 2 h with *P. gingivalis* LPS stimulation. In addition, NLRP3 was significantly up-regulated after pre-incubation with ATP for 3 h then infection with *P. gingivalis* and stimulation with *P. gingivalis* LPS for 2 h, but was down-regulated at 4 h. At the protein level (Fig. 2), while cells were incubated for 2 and 4 h with *P. gingivalis* and *P. gingivalis* LPS stimulation or pretreatment with ATP for 3 h, the trends of the proteolysis of NLRP3 bands corresponded to that of the gene expression. This indicated that the activation of NLRP3 depended on *P. gingivalis* LPS or/and ATP, but not *P. gingivalis* infection.

In Fig.1b, during the time course of *P. gingivalis* infection, there were significant decreases in ASC mRNA levels (2 and 4 h) compared with the control group, and significant increases of ASC mRNA levels were observed at 2 h stimulation with *P. gingivalis* LPS. For ATP plus *P. gingivalis* infection, there was up-regulation of ASC at 2 h, followed by down-regulation at 4 h compared with the ATP pre-treatment group. For ATP plus *P. gingivalis* LPS stimulation, there was a marked reduction of ASC at 4 h compared with the ATP pre-treatment group. These results suggest that ASC mRNA changes are critical in *P. gingivalis* LPS and ATP induced NLRP3 inflammasome activation. At the protein level, similar changes in ASC protein were measured (data not shown).

Additionally, as evident in Fig. 1c, the data showed that there was no increase in caspase-1 gene expression in both *P. gingivalis* infected and *P. gingivalis* LPS stimulated groups. After cells were pre-incubated with ATP for 3 h, the caspase-1 level in both *P. gingivalis* infection (2 h) and *P. gingivalis* LPS stimulation (2 and 4 h) groups was significant up-regulated compared with the ATP pretreatment group. These results indicate that caspase-1 activation depends on ATP stimulation in *P. gingivalis* infected or *P. gingivalis* LPS treated cells. There were no changes on caspase-1 protein level by immunoblots (data not shown).

### IL-1β expression in response to *P. gingivalis*, *P. gingivalis* LPS and ATP plus *P. gingivalis*/*P. gingivalis* LPS

**Fig. 3:**
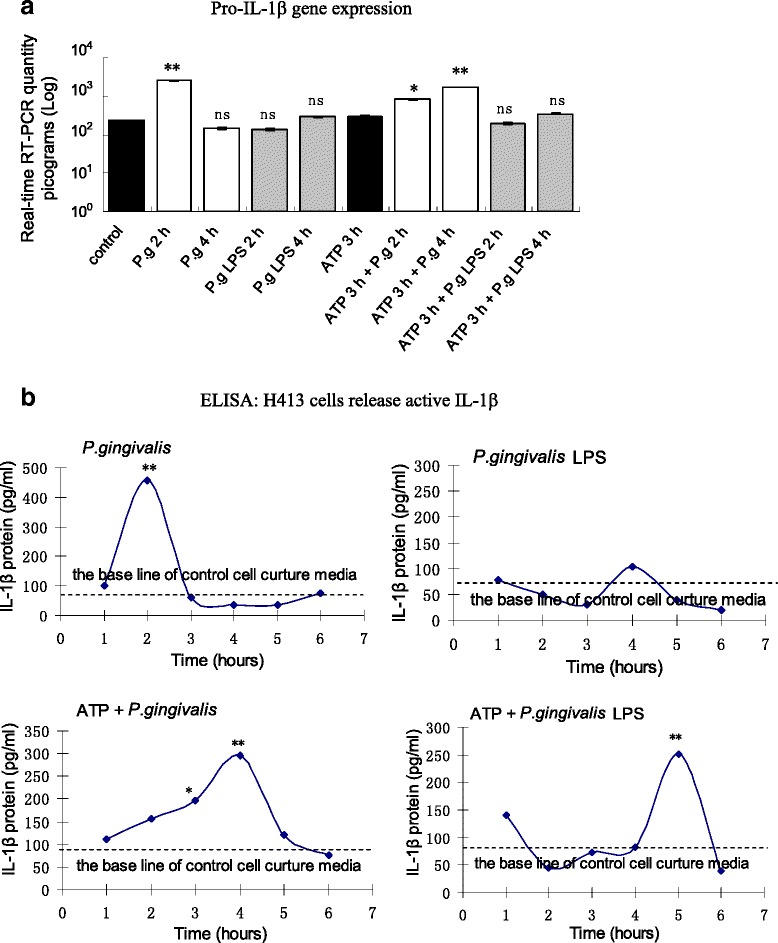
**a** Changes in pro-IL-1β mRNA levels in H413 cells treated with *P. gingivalis* infection and ATP pretreatment. **b** ELISA data showing mature IL-1β protein released from H413 cells after different treatments. * *P* < 0.05, ** *P* < 0.01, paired *t*-test

Similarly, to determine changes in IL-1β expression through inflammasome components activation, the gene expression of IL-1β was measured by real-time RT-PCR (Fig. 3a). During the time course of *P. gingivalis* infection, a significant increase in pro-IL-1β mRNA level was measured at 2 h compared with the control group, which then decreased to the control level at 4 h. Increased pro-IL-1β mRNA levels were also observed after cells pre-treated with ATP for 3 h then infected by *P. gingivalis* at 2 and 4 h. There were no changes with cells stimulated with *P. gingivalis* LPS and ATP plus *P. gingivalis* LPS. In cell culture supernatants (Fig. 3b), there was a high concentration of mature IL-1β at 2 h in the *P. gingivalis* infection group, and at 3 and 4 h in the ATP plus *P. gingivalis* infection group, which corresponded with pro-IL-1β gene expression (Fig. 3a). There was no secretion of IL-1β detected in the *P. gingivalis* LPS stimulation group, but a notable delayed increase in response to ATP plus *P. gingivalis* LPS group at 5 h (Fig. 3b). These results indicated that *P. gingivalis* infection has a greater capacity to induce IL-1β secretion than *P. gingivalis* LPS.

### IL-18 expression in response to in response to *P. gingivalis*, *P. gingivalis* LPS and ATP plus *P. gingivalis*/*P. gingivalis* LPS

**Fig. 4:**
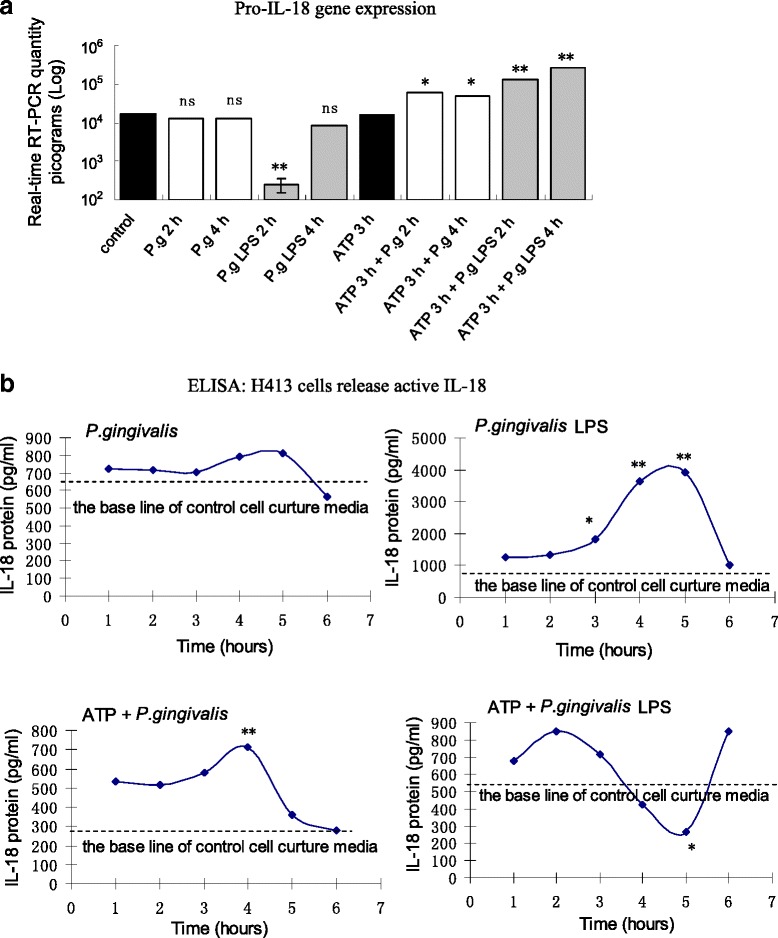
**a** Changes in pro-IL-18 mRNA levels in H413 cells with different treatments of *P. gingivalis* LPS stimuli and ATP plus *P. gingivalis* infection or *P. gingivalis* LPS. **b** ELISA data showing mature IL-18 protein released from H413 cells after different treatments. * *P* < 0.05, ** *P* < 0.01, paired *t*-test

The gene expression of IL-18 was also measured by real-time RT-PCR (Fig. 4a). There was no change in pro-IL-18 measured in the *P. gingivalis* infection group, but significantly decreased pro-IL-18 in the *P. gingivalis* LPS stimuli group at 2 h. In addition, the pre-treatment of cells with ATP for 3 h induced the up-regulation of pro-IL-18 at all time points and significantly increased IL-18 expression in both the *P. gingivalis* infection and *P. gingivalis* LPS stimuli groups. In cell culture supernatants (Fig. 4b), cells stimulated with *P. gingivalis* LPS had a significant increase in mature IL-18 secretion which reached a peak between 4 h and 5 h. This was not evident in the *P. gingivalis* infection group. This high protein level did not correspond with the low pro-IL-18 mRNA level in the *P. gingivalis* LPS group, possibly as protein concentration is affected by several parameters - mainly synthesis and cleavage [34]. After cells were pre-treated with ATP for 3 h, secretion of mature IL-18 into cell culture media was measured in the ATP plus *P. gingivalis* infection group, and demonstrated a notable increase in cytokine concentration. However, in the ATP plus *P. gingivalis* LPS group, a biphasic curve was shown, which did not reflect the mRNA level [34]. These results indicate that *P. gingivalis* infection did not induce mature IL-18 secretion, while *P. gingivalis* LPS or ATP stimulation led to a mature IL-18 production.

### Changes in the NLRP3 inflammasome complex after stimulation with *P. gingivalis*, *P. gingivalis* LPS and ATP

**Fig. 5:**
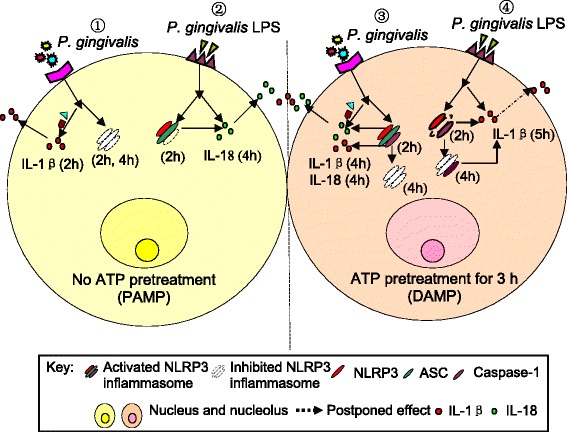
Innate immune response to *P. gingivalis* infection, or *P. gingivalis* LPS and ATP stimulation in epithelial cells. In the left panel, when cells are stimulated with *P. gingivalis* infection or *P. gingivalis* LPS without ATP pretreatment, the NLRP3 inflammasome complex is inhibited until *P. gingivalis* LPS stimulation, except for caspase-1. IL-1β production is not NLRP3 inflammasome dependent, however IL-18 secretion relies on NLRP3 and ASC activation. In the right panel, cells pretreated with ATP, which activates NLRP3 inflammasome, results in the subsequent release of the cytokines IL-1β and IL-18 in *P. gingivalis* infected cells. ATP can also promote caspase-1 activation upon *P. gingivalis* LPS stimulation. Secretion of IL-1β is always consistent with caspase-1 activation after stimulation with *P. gingivalis* LPS

Figure 5 shows a summary of the innate immune response against *P. gingivalis* infection, and *P. gingivalis* LPS and ATP stimulation in this cell model. When cells are not pretreated with ATP (left panel), infection with *P. gingivalis* in this model does not trigger the activation of the NLRP3 inflammasome complex, including NLRP3, ASC and caspase-1, until *P. gingivalis* LPS stimulation, even though in a short time 2 h (except caspase-1). Notably, IL-1β production did not rely on activation of the NLRP3 inflammasome and is involved in the early stages of inflammation. IL-18 production requires both NLRP3 and ASC activation after stimulation with *P. gingivalis* LPS. When cells are pretreated with ATP (right panel), a rapid response of activation of NLRP3 inflammasome was shown (2 h), with subsequent production of the cytokines IL-1 β and IL-18 in cells with *P. gingivalis* infection. However, when cells are stimulated with *P. gingivalis* LPS, IL-1β production required caspase-1 activation and showed a delayed increase in response to ATP treatment (right panel). The results indicate that *P. gingivalis*, *P. gingivalis* LPS and ATP all have different effects on epithelial cells to produce the final inflammatory response.

## Discussion

In this study, *P. gingivalis* infection down-regulates NLRP3 inflammasome components, including NLRP3, ASC and caspase-1 in epithelial cells at all time points, which indicates that *P. gingivalis* may either up-regulate or down-regulate NLRP3 expression, depending on the cell type [35]. In this model, *P. gingivalis* may dampen the endpoint innate immune responses by inhibiting the activation of NLRP3 inflammasome and evading host surveillance, offering a survival advantage to all co-habiting organisms of the biofilm [35]. Meanwhile, up-regulated gene expression of IL-1β after *P. gingivalis* infection, demonstrates that IL-1β is a critical cytokine in the host defence against *P. gingivalis* infection [5]. Moreover, IL-1β is not NLRP3 or caspase-1 dependent [5, 36] and other factors may also influence the IL-1β protein secretion. Therefore, we speculate that the secretion of IL-1β in the early stages of *P. gingivalis* infection would play a very important role in combating the invading pathogen as part of the innate immune response [37]. This is consistent with Dinarello’s conclusion that IL-1β is one of the earliest cytokines to be secreted during the initial phases of inflammation and participates in almost all events involved in the activation and regulation of inflammation [36]. This kind of inflammasome-independent IL-1β activation can substantially contribute to tissue inflammation [38].

In the present study, LPS elicits a striking immune response through up-regulation of the gene expression of NLRP3 and ASC, but not caspase-1, which indicates that *P. gingivalis* LPS would be a key factor in eliciting the inflammatory response that leads to the diseased state [16] and is considered an important virulence factor in the pathogenesis of periodontal disease. However, Jain and Darveau’s research has elucidated that activation of NLRP3 and ASC after proinflammatory stimuli such as LPS may be involved in the apoptosis of host cells, which supports the idea of *P. gingivalis*-induced cell death [39], which would then facilitate periodonto-pathogens to invade and destroy epithelial tissues. In addition, caspase-1 is synthesised as an inactive zymogen. Its activation is tightly regulated by inflammasomes and associated with a rapid and lytic form of cell death known as pyroptosis [40]. This could explain the mechanism that caspase-1 activation is inhibited after *P. gingivalis* LPS stimulation until activated with ATP stimuli in the present study.

ATP is a very efficient extracellular distress signal [41]. As one of the first activators described to induce NLRP3 inflammasome formation, it is ascribed to the group of endogenous DAMPs, which come from dying cells [22]. In this study, we demonstrate that ATP activates the NLRP3 inflammasome, subsequently releasing cytokines IL-1 β and IL-18, even though the effect is very transient and the concentration may not be high enough to maintain the level of activated NLRP3 inflammasome. These findings support that extracellular ATP, as a danger signal, results in assembly of NLRP3 inflammasome and secretion of mature cytokines in *P. gingivalis*-infected cells [26]. In addition, a common denominator of extracellular ATP activating NLRP3 has the ability to form membrane pores that induce damage of membrane integrity or cause perturbation of the intracellular ion concentration [22].

Moreover, after stimulation with ATP and *P. gingivalis* LPS, the reduction of ASC gene expression may serve as a mechanism for shutting down inflammation, thus avoiding overzealous immune responses [32]. Similarly, the epithelial cell line (H413) used in this study has the function to restrain the activity and secretion of IL-1β [42] to protect the cell against the tissue destruction. This can explain our finding showing a postponed increase of IL-1β in response to ATP plus *P. gingivalis* LPS stimulation.

## Conclusions

The findings indicate that *P. gingivalis* LPS stimulation induced a greater proinflammatory reaction than *P. gingivalis* infection, and this action becomes more intense after pre-treatment of ATP. These results may provide new insights into targets for therapeutic strategies to treat inflammatory diseases such as periodontitis.

## Abbreviations

ASC: Apoptosis-associated speck-like protein
ATP: Adenosine triphosphate
DAMPs: Danger-associated molecular patterns
NLRP3: Nod-like receptor (NLR) family, the pyrin domain-containing-3
PAMPs: Pathogen-associated molecular patterns
*P. gingivalis*: *Porphyromonas gingivalis*
*P. gingivalis* LPS: Lipopolysaccharide from *P. gingivalis*.
